# Scores based on neutrophil percentage and lactate dehydrogenase with or without oxygen saturation predict hospital mortality risk in severe COVID-19 patients

**DOI:** 10.1186/s12985-021-01538-8

**Published:** 2021-03-31

**Authors:** Xiude Fan, Bin Zhu, Masoud Nouri-Vaskeh, Chunguo Jiang, Xiaokai Feng, Kyle Poulsen, Behzad Baradaran, Jiansong Fang, Erfan Ahmadi Ade, Akbar Sharifi, Zhigang Zhao, Qunying Han, Yong Zhang, Liming Zhang, Zhengwen Liu

**Affiliations:** 1 Department of Infectious Diseases, First Affiliated Hospital of Xi’an Jiaotong University, No. 277 Yanta West Road, Xi’an, 710061 Shaanxi Province People’s Republic of China; 2 Department of Pharmacy, Beijing Tiantan Hospital, Capital Medical University, Beijing, China; 3 Immunology Research Center, Tabriz University of Medical Sciences, Tabriz, Iran; 4 Department of Respiratory and Critical Care Medicine, Beijing Institute of Respiratory Medicine, Beijing Chaoyang Hospital, Capital Medical University, Beijing, People’s Republic of China; 5 Department of Inflammation and Immunity, Cleveland Clinic, Cleveland, OH USA; 6 Science and Technology Innovation Center, Guangzhou University of Chinese Medicine, Guangzhou, Guangdong Province China; 7 DME Center, Institute of Clinical Pharmacology, Guangzhou University of Chinese Medicine, Guangzhou, Guangdong Province People’s Republic of China; 8 Tuberculosis and Lung Disease Research Center, Tabriz University of Medical Sciences, Tabriz, Iran; 9 Department of Hepatobiliary Surgery, Union Hospital, Tongji Medical College, Huazhong University of Science and Technology, Wuhan, People’s Republic of China

**Keywords:** Severe COVID-19, SARS-CoV-2, Hospital mortality, Prediction

## Abstract

**Background:**

Risk scores are needed to predict the risk of death in severe coronavirus disease 2019 (COVID-19) patients in the context of rapid disease progression.

**Methods:**

Using data from China (training dataset, n = 96), prediction models were developed by logistic regression and then risk scores were established. Leave-one-out cross validation was used for internal validation and data from Iran (test dataset, n = 43) was used for external validation.

**Results:**

A NSL model (area under the curve (AUC) 0.932) and a NL model (AUC 0.903) were developed based on neutrophil percentage and lactate dehydrogenase with and without oxygen saturation (SaO_2_) using the training dataset. AUCs of the NSL and NL models in the test dataset were 0.910 and 0.871, respectively. The risk scoring systems corresponding to these two models were established. The AUCs of the NSL and NL scores in the training dataset were 0.928 and 0.901, respectively. At the optimal cut-off value of NSL score, the sensitivity and specificity were 94% and 82%, respectively. The sensitivity and specificity of NL score were 94% and 75%, respectively.

**Conclusions:**

These scores may be used to predict the risk of death in severe COVID-19 patients and the NL score could be used in regions where patients' SaO_2_ cannot be tested.

**Supplementary Information:**

The online version contains supplementary material available at 10.1186/s12985-021-01538-8.

## Background

Coronavirus disease 2019 (COVID-19), caused by severe acute respiratory syndrome coronavirus-2 (SARS-CoV-2), is a highly contagious and fast-spreading infectious disease. It constitutes a pandemic within only ten months and is spreading in many countries worldwide with millions of people being affected [1].

The clinical spectrum of COVID-19 ranges from mild to critically ill diseases according to the largest cohort study (44,672 persons with COVID-19) from China [2]. COVID-19 can progress rapidly into acute respiratory distress syndrome (ARDS), multiorgan failure, and even death during the later stages in some severe cases [2–6]. Clinicians should be aware that some patients may deteriorate rapidly after admission.

Since the outbreak of COVID-19, researchers and clinicians are acting quickly, but it is difficult to make meaningful progress compared to the progression and variation rate of this disease. Unfortunately, clinically useful indexes to predict the disease prognosis, especially for severe cases, remain unavailable. Previous studies have identified that lymphopenia, neutrophilia, elevated serum alanine aminotransferase (ALT), aspartate aminotransferase levels (AST), lactate dehydrogenase (LDH), D-dimer and C-reactive protein (CRP) all may be associated with disease progression and death [3–5, 7, 8]. However, there is no easy-to-use risk-scoring system for the risk of death in severe patients. Currently, clinicians urgently need a convenient risk assessment tool to assist them in predicting the risk of hospital mortality in patients with COVID-19. Such a tool would allow clinicians to select the optimal timing and method of medical intervention for patients and to evaluate the effectiveness of treatment strategies.

Therefore, in the current study, we aimed to establish straightforward and user-friendly prediction models to predict the risk of in-hospital death in severe patients with COVID-19, using data from patients with confirmed severe COVID-19 who were admitted to hospitals in China and Iran.

## Methods

### Patient population

**Fig. 1:**
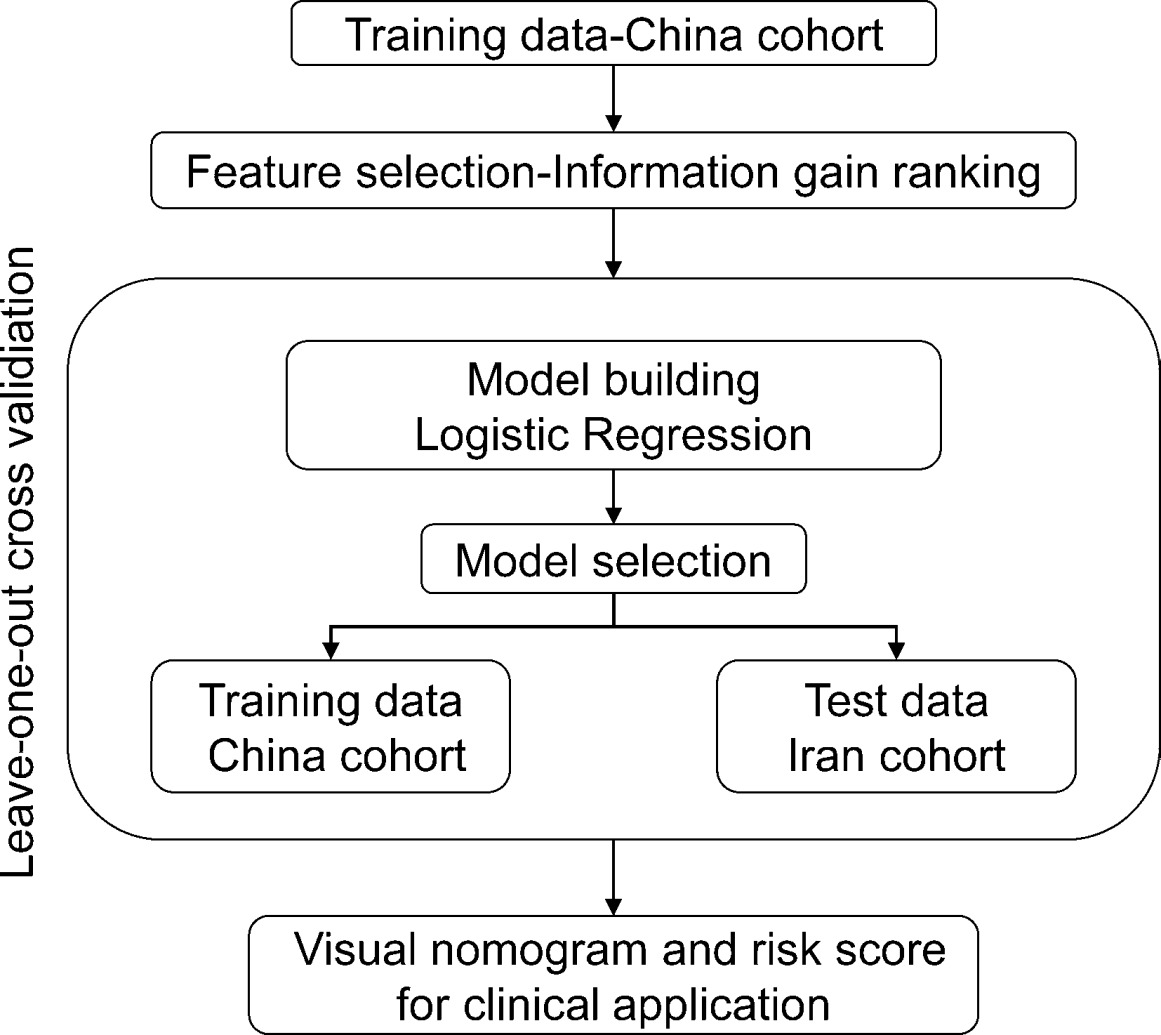
Flow chart of the study population selection and analysis method

This multicentric retrospective observational study was based on two datasets of severe patients with confirmed SARS-CoV-2 infection selected by the same criteria [9] from 2 medical centers (West Branch of Union Hospital; Tongji Medical College of Huazhong University of Science and Technology in China and Tabriz University of Medical Sciences in Iran). The patients’ data from China were used as the training dataset to establish models for predicting the risk of hospital mortality, whereas the patients’ data from Iran was used for external validation of the prediction models (Fig. 1). All severe patients with confirmed SARS-CoV-2 infection in the training and test datasets were included if they were adults. Pregnant patients and patients with human immunodeficiency virus infection were excluded.

This study was approved by the Ethics Committees of two participating hospitals in China (Union Hospital, affiliated with Tongji Medical College, Huazhong University of Science and Technology) and Iran (Tabriz University of Medical Sciences, approval number: IR. TBZMED. REC.1399.008).

### Data collection

We reviewed clinical medical records, nursing records, and laboratory examinations for all severe patients with laboratory-confirmed SARS-CoV-2 infection. The severity of disease was classified according to Chinese Clinical Guidance for COVID-19 Pneumonia Diagnosis and Treatment (7th edition) [9]. We collected admission data of these patients including age, sex, symptoms (fever, cough, sputum, fatigue, shortness of breath, headache, and diarrhea), medical histories (chronic cardiovascular disease, chronic pulmonary disease, cerebrovascular disease, diabetes, malignancy, chronic liver and kidney disease and smoking history), signs and symptoms (heart rate, respiratory rate, and oxygen saturation (SaO_2_)), laboratory indexes (white blood cells (WBC), neutrophil percentage (NE), lymphocyte percentage (LY), hemoglobin (HGB), hematocrit (HCT), platelets (PLT), LDH, total bilirubin (Tbil), direct bilirubin (Dbil), ALT, AST, total protein, albumin (ALB), activated partial thromboplastin time (APTT), prothrombin time (PT), D-dimer, CRP, blood urea nitrogen (BUN), serum creatinine (Cr), creatinine clearance (CCr), blood glucose, creatine kinase isoenzymes (CKMB), high density lipoprotein (HDL), low density lipoprotein (LDL), total cholesterol (TC), triglyceride (TG), Lipoprotein, Apolipoprotein A (ApoA), Apolipoprotein B (ApoB), serum potassium (K), and serum sodium (Na)). HDL, LDL, TC, TG, Lipoprotein, ApoA, ApoB, HGB, and HCT were not collected in the Iranian population. Information about treatment during hospitalization (antiviral therapy, antibacterial therapy, corticosteroids, and immunoglobulin therapy) and outcome (in-hospital death) were also collected.

### Statistical analysis

Continuous variables are reported as means ± standard error (SE). Unpaired t-test or the Mann–Whitney test was used to compare two groups of data. Categorical variables are expressed as counts and percentages; Chi-square or Fisher's exact tests were used for comparisons of categorical factors. Feature selection was performed to select the suitable variables to establish the prognostic model using the information gain method. Information gain was calculated by comparing the entropy of the data before and after transformation [10]. Factors with attributes of variables > 0.2 were selected for modeling. The establishment of death risk models was based on multivariable logistic regression models using training dataset. The predictive accuracy for the prognostic accuracy of hospital mortality of severe patients was calculated using receiver operating characteristic (ROC) curves. When the sensitivity, specificity and area under the curve (AUC) were basically similar between different models, we selected models for further analysis based on the premise of minimizing the number of factors included in the model. Validity assessment of the predictive models was conducted using internal and external validation. We used leave-one-out cross-validation method for internal validation to limit model over-fitting and to assess predictive potential [11]. In external validation, models developed in the training dataset were applied on the test dataset to assess the predictive performance of models. We used calibration plots to show the goodness-of-fit of models and plotted nomograms to facilitate the clinical application of both models. The Hosmer–Lemeshow tests were also used to assess model goodness-of-fit. In addition, in order to simplify the computation of in-hospital death risk estimate, we developed risk scores based on the points system from the Framingham Heart Study methodology [12]. First, continuous variables (LDH, NE, and SaO_2_) were converted to categories and reference values for each variable were separately defined. Second, we determined the referent predictive factor profile (WiREF) by assigning the median value in each category and calculated the difference values between each category and the reference value (Wij-WiREF). Third, beta regression coefficients (Bi) for continuous variables (LDH, NE, and SaO_2_) were obtained. The point score for each category of predictors was estimated using the product of the corresponding beta regression coefficients (Bi) and the difference values between each category (Wij-WiREF), and the reference value (B). The point range was calculated based on the points for each predictor. Once the simple point system was generated, we evaluated its diagnostic capacity in the train and test cohorts using ROC curves. The optimal cut-off values for ROC curves were established using the Youden index. All statistical analyses were performed using STATA (Version 13.0, IBM, New York, USA) and Orange (Version 3.24.1, USA).

## Results

### Characteristics of the study population

There were 96 patients from China in the training dataset and 43 patients from Iran in the test dataset. The mean age of patients in the training and test datasets were 63.47 and 63.37 years, respectively. The patients in the two datasets differ in several characteristics at the time of admission (Table 1). In total, there are 49 (51%) male patients in the training and 30 (69.8%) male patients in the test dataset (*P* = 0.039). There were more patients with fever (89.6% versus 46.5%), fatigue (89.6% versus 42.2%) and diarrhea (20.8% versus 2.3%) in the training dataset compared to those in test dataset. In addition, patients in the training dataset had faster respiratory rates (27.24 versus 22.76) than those in the test dataset. The proportion of deaths in the two data sets (32.3% versus 30.2%) was roughly the same.

**Table 1:** Clinical characteristics of the severe patients with COVID-19

Variables	Training dataset (China data) n = 96	Test dataset (Iran data) n = 43	*P* value
Age (years), mean (SE)	63.47 (1.36)	63.37 (2.70)	0.972
Male, n (%)	49 (51.0)	30 (69.8)	0.039
Smoking history, n (%)	1 (1.0)	2 (4.7)	0.064
Symptoms on admission, n (%)			
Fever	86 (89.6)	20 (46.5)	< 0.001
Cough	78 (81.3)	23 (53.5)	0.222
Fatigue	86 (89.6)	27 (42.2)	< 0.001
Shortness of breath	70 (72.9)	24 (55.8)	0.983
Headache	17 (17.7)	4 (9.3)	0.453
Diarrhea	20 (20.8)	1 (2.3)	0.017
Coexisting disorder, n (%)			
Hypertension	33 (34.4)	12 (27.9)	0.748
Diabetes	16 (16.7)	8 (18.6)	0.296
Chronic obstructive pulmonary disease	3 (3.1)	2 (4.7)	0.444
Cerebral infarction	1 (1.0)	1 (2.3)	0.42
Coronary heart disease	10 (10.4)	0	0.057
Chronic kidney disease	3 (3.1)	1 (2.3)	0.768
Chronic liver disease	0	0	–
Respiratory rate, mean (SE)	27.24 (0.57)	22.76 (1.09)	< 0.001
Heart rate, mean (SE)	92.89 (1.749)	90.34 (1.88)	0.323
In-hospital deaths, n (%)	31 (32.3)	13 (30.2)	0.809

### Feature selection

**Fig. 2:**
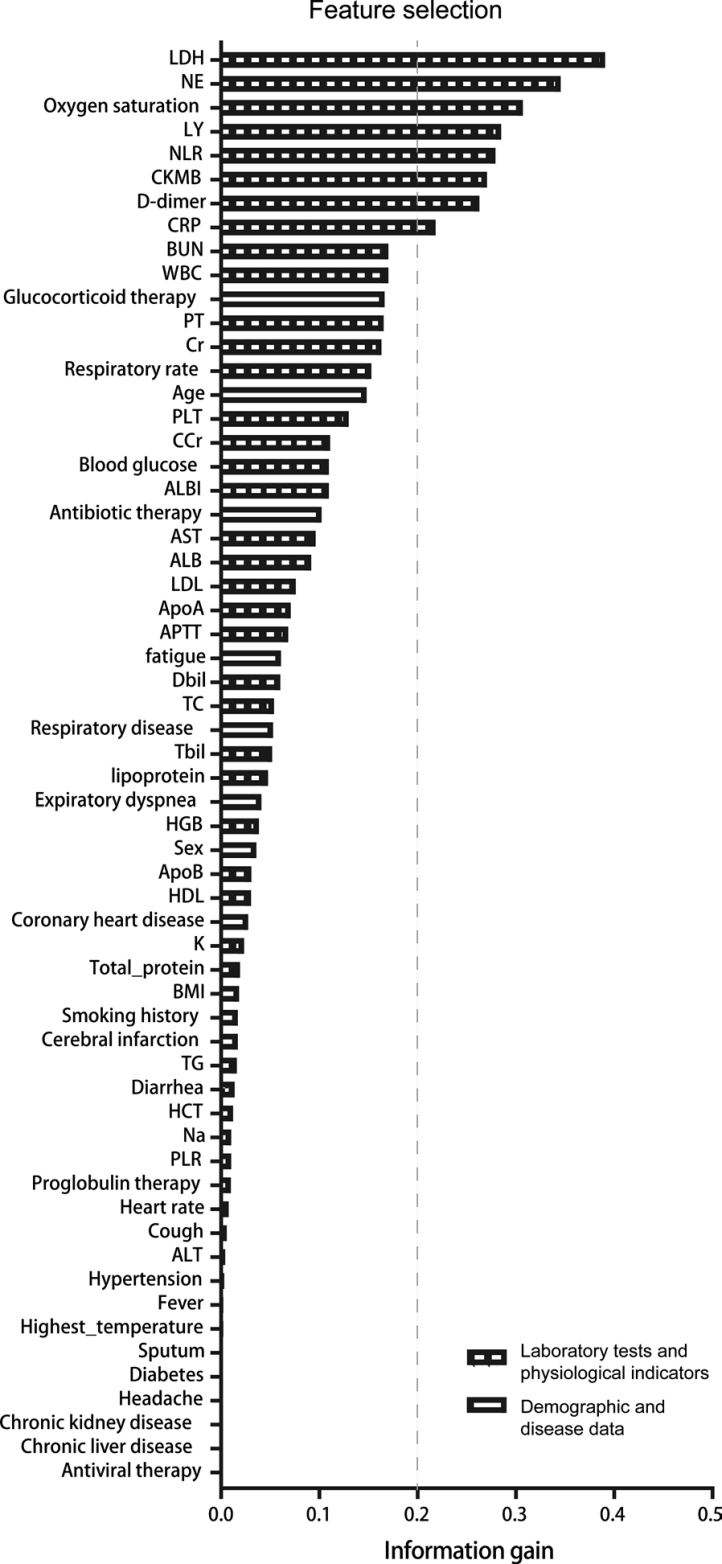
Feature selection to find variables with respect to the hospital mortality of severe patients. *SaO*_*2*_ oxygen saturation, *WBC* white blood cells, *NE* neutrophil percentage, *LY* lymphocyte percentage, *NLR* neutrophils/lymphocytes ratio, *HGB* hemoglobin, *HCT* hematocrit, *PLT* platelets, *LDH* lactate dehydrogenase, *Tbil* total bilirubin, *Dbil* direct bilirubin, *ALT* alanine aminotransferase, *AST* aspartate amino transferase, *ALB* albumin, *APTT* activated partial thromboplastin time, *PT* prothrombin time, *CRP* C-reactive protein, *BUN*, blood urea nitrogen, *Cr* serum creatinine, *CCr* creatinine clearance, *CKMB* creatine kinase isoenzymes, *HDL* high density lipoprotein, *LDL* low density lipoprotein, *TC* total cholesterol, *TG* triglyceride, *ApoA* Apolipoprotein A, *ApoB* Apolipoprotein B, *K* serum potassium, *Na* serum sodium

Figure 2 shows the results from information gain ranking, the top 8 (information gain > 0.2) of the available 60 variables (LDH, NE, SaO_2_, LY, NLR, CKMB, D-dimer, and CRP) were selected for modeling according to the criteria. As shown in Additional file 1: Fig. S1A, LDH, NE, NLR, CKMB, D-dimer, and CRP were significantly higher and SaO_2_, and LY were lower in the severe patients who died during hospitalization compared to patients who did not die.

### Derivation and validation of NSL model and NL model

When used individually to predict the risk of death, AUCs of top 8 ranked variables range from 0.763 to 0.880, sensitivities ranged from 73 to 100%, and specificities ranged from 51 to 88% (Table 2). Each of these indicators had a good prediction ability for the risk of death, but there were some exceptions, such as some patients with normal indicators who also died during hospitalization. Therefore, integrated prediction models were needed to reduce the defects of a single indicator in predicting death risk.

**Table 2:** Predictive capacity of the factors and integrated models for the risk of hospital mortality in severe patients with COVID-19

Variable	AUC	95%CI	SE	Sensitivity (%)	Specificity (%)
Training data from China cohort					
LDH (U/L)	0.880	0.813–0.948	0.034	97	71
NE (%)	0.879	0.812–0.946	0.034	84	82
SaO_2_ (%)	0.849	0.758–0.940	0.046	87	78
LY (%)	0.852	0.776–0.929	0.039	77	80
NLR	0.858	0.783–0.933	0.038	81	82
CKMB (U/L)	0.829	0.746–0.912	0.042	87	69
D-dimer (μg/mL)	0.763	0.641–0.885	0.062	73	88
CRP (μg/mL)	0.807	0.723–0.892	0.043	100	51
*Integrated models*					
All variables with information gain > 0.2					
LDH + NE + SaO_2_ + LY + NLR + CKMB + D-dimer + CRP	0.945	0.897–0.992	0.024	97	83
NE was selected for modeling					
LDH + NE + SaO_2_ + CKMB + D-dimer + CRP	0.945	0.900–0.989	0.023	93	84
LDH + NE + SaO_2_ + CKMB + D-dimer	0.942	0.898–0.987	0.023	97	78
LDH + NE + SaO_2_ + CKMB	0.937	0.887–0.988	0.026	83	94
LDH + NE + SaO_2_ (NSL risk score)	0.932	0.884–0.981	0.025	97	78
LDH + NE (NL risk score)	0.903	0.843–0.963	0.031	94	82
LY was selected for modeling					
LDH + SaO_2_ + LY + CKMB + D-dimer + CRP	0.948	0.904–0.992	0.022	97	84
LDH + SaO_2_ + LY + CKMB + D-dimer	0.944	0.901–0.987	0.022	86	88
LDH + SaO_2_ + LY + CKMB	0.932	0.880–0.984	0.026	97	77
LDH + SaO_2_ + LY	0.934	0.886–0.982	0.025	90	88
LDH + LY	0.903	0.843–0.964	0.031	90	82
NLR was selected for modeling					
LDH + SaO_2_ + NLR + CKMB + D-dimer + CRP	0.930	0.866–0.995	0.033	83	95
LDH + SaO_2_ + NLR + CKMB + D-dimer	0.945	0.901–0.989	0.022	79	95
LDH + SaO_2_ + NLR + CKMB	0.933	0.882–0.983	0.026	77	97
LDH + SaO_2_ + NLR	0.933	0.883–0.971	0.025	87	88
LDH + NLR	0.919	0.866–0.971	0.027	90	82
LDH wasn’t selected for modeling					
NE + SaO_2_	0.919	0.865–0.972	0.027	97	78
Test data from Iran cohort					
LDH (U/L)	0.746	(0.574–0.919)	0.0881	77	70
NE (%)	0.851	0.719–0.984	0.068	85	82
SaO_2_ (%)	0.869	0.702–1.000	0.085	85	97
Combined models					
LDH + NE + SaO_2_ (NSL risk score)	0.910	0.758–1.000	0.077	92	96
LDH + NE (NL risk score)	0.871	0.734–1.000	0.071	92	82

*LDH* lactate dehydrogenase, *NE* neutrophil percentage, *SaO*_*2*_ oxygen saturation, *LY* lymphocyte percentage, *NLR* neutrophils/lymphocytes ratio, *CKMB* creatine kinase myocardial bound, *CRP* C-reactive protein, *AUC* area under the curve

In the modeling, we tried to use as few variables as possible to facilitate clinical application. Because the NE and LY had a reciprocal relationship and integrated models were based on the logistic regression method, we established three model groups depending on whether the NE, LY, or neutrophils/lymphocytes ratio (NLR) was added to the model. AUCs of all integrated models ranged from 0.903 to 0.948, sensitivities ranged from 77 to 97%, and specificities ranged from 77 to 97% (Table 2). The integrated model combined all top 8 variables (AUC 0.945; sensitivity 97% and specificity 83%), the NSL model combinied NE, SaO_2_ and LDH (AUC 0.932; sensitivity 97% and specificity 78%; Additional file 1: Fig. S1b), and the NL model combined 2 variables, NE and LDH (AUC 0.903; sensitivity 94% and specificity 82%; Additional file 1: Fig. 1b) all had high sensitivity and specificity in predicting the risk of death. Considering the need for convenient clinical application and the regions with less-advanced medical care level, we selected the NSL model and NL model for validation in the test dataset. The NL model could be used in regions where patients’ SaO_2_ concentrations cannot be tested regularly.

Compared with the training dataset, the NSL model (AUC 0.910; sensitivity 92% and specificity 96%) and NL model (AUC 0.871; sensitivity 92% and specificity 82%) both provided similarly accurate predictability of in-hospital death in the test dataset (Table 2 and Additional file 1: Fig. 1c).

### Nomogram prediction for in-hospital death of severe patients

**Fig. 3:**
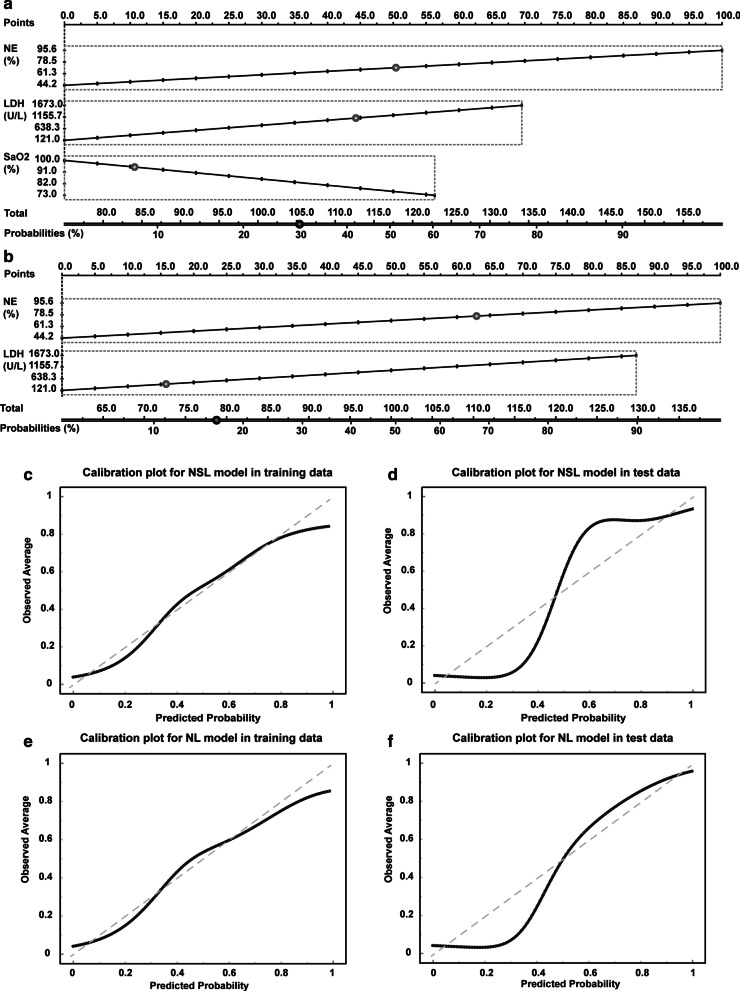
Nomograms for integrated models to predict hospital mortality and d the corresponding calibration plots. Nomgrams of the NSL model (**a**) and NL model (**b**) to estimate the risk of death in severe patients with COVID-19. Calibration plot showing the probability of death. Plots for NSL model in training (**c**) and test dataset (**d**). Calibration plots for NL model in training (**e**) and test dataset (**f**). The nomogram-estimated mortality is plotted on the x-axis, and the actual mortality is plotted on the y-axis. The diagonal dotted line is a perfect estimation by an ideal model. The solid lines are the performance of the nomogram, and closer alignment with the dashed diagonal lines indicates a better estimate

In order for clinicians to easily calculate the risk of mortality using the NSL model or NL model, we created two nomograms to provide graphical depictions of all indicators in the NSL and NL models, respectively (Fig. 3a, b). In both the training and test datasets, the calibration plots of nomograms were consistent between the predicted risk and the observed probability of death (Fig. 3c–f). The Hosmer–Lemeshow tests for NSL model and NL model were not significant (*P* = 0.47 and *P* = 0.45), suggesting the NSL model and NL model were correctly specified for the prediction of in-hospital death from COVID-19.

### Development of risk scoring system for predicting in-hospital death

In addition to providing a nomogram to help clinicians predict the mortality risk of severe patients, we also developed two risk scoring systems based on NSL model and NL model. As shown in Table 3, simple point systems were developed based on the logistic regression coefficients (Additional file 1: Table S1) and reference values for each significant risk factor (Table 3). The NSL risk score included NE (16 points), SaO_2_ (9 points), and LDH (9 points). The total points ranged from 0 to 34, and with increasing total points, the risk of death increased. Points of 0–13 were associated with a less than 10% risk of death, points of 14–20 with a 10–50% risk of death, and points above 20 were associated with an extremely high risk of death over 50%. The cut-off of the NSL risk score for the prediction of death in training dataset is 15 (sensitivity 94% and specificity 82%, Additional file 1: Table S2). The AUCs of the NSL risk score were 0.928 and 0.901 in the training and test dataset, respectively. In addition, the NL risk score included NE (16 points) and LDH (9 points). The score ranged from 0 to 25. The AUCs of the NL risk score were 0.895 and 0.857 in the training and test dataset, respectively. Points of 0–9 were associated with a less than 10% risk of death, points of 10–15 with a 10–50% risk of death, and points above 16 were associated with an extremely high risk of death over 50%. The cut-off of the NL risk score for the prediction of death in training dataset is 12 (sensitivity 94% and specificity 75%, Additional file 1: Table S2). In clinical practice, clinicians can calculate the risk scores of each patient at admission based on the points provided in Tables 3 and 4.

**Table 3:** Algorithm to estimate risk for hospital mortality using total points for risk scores with logistic regression analysis in the severe patients with COVID-19 from training dataset

Variables	Categories	Reference value (W_ij_)	Bi	Regression units Βi (W_ij_—W_iREF_)	Points assigned Βi (W_ij_—W_iREF_)/B
NSL risk score (NE + SaO_2_ + LDH)					
NE (%)			0.127		
	≤ 60	55 (W_iREF_)		0.000	0
	60.1–70	65		1.270	4
	70.1–80	75		2.540	8
	80.1–90	85		3.810	12
	≥ 90.1	95		5.080	16
SaO_2_ (%)			-0.175		
	100–96	98 (W_iREF_)		0.000	0
	95–91	93		0.875	3
	90–86	88		1.750	6
	≤ 85	83		2.625	9
LDH (U/L)			0.003		
	≤ 221	171 (W_iREF_)		0.000	0
	222–321	271		0.300	1
	322–421	371		0.600	2
	422–521	471		0.900	3
	522–621	571		1.200	4
	622–721	671		1.500	5
	722–821	771		1.800	6
	822–921	871		2.100	7
	922–1021	971		2.400	8
	≥ 1022	1071		2.700	9
NL risk score (NE + LDH)					
NE (%)			0.158		
	≤ 60	55 (W_iREF_)		0.000	0
	60.1–70	65		1.580	4
	70.1–80	75		3.160	8
	80.1–90	85		4.740	12
	≥ 90.1	95		6.320	16
LDH (U/L)			0.004		
	≤ 221	171 (W_iREF_)		0	0
	222–321	271		0.400	1
	322–421	371		0.800	2
	422–521	471		1.200	3
	522–621	571		1.600	4
	622–721	671		2.000	5
	722–821	771		2.400	6
	822–921	871		2.800	7
	922–1021	971		3.200	8
	≥ 1022	1071		3.600	9

Wij, reference value for each category of risk factors in risk score; WiREF, the base category for each risk factor was used as the basic value for that factor and assigned 0 point. Bi, the regression coeffcient of each risk factor from logistic regression; B, the smallest regression units or the smallest units divided by some constant (B = 0.3 for NSL risk score and B = 0.4 for NL risk score)

**Table 4:** The risk of in-hospital death corresponding to the sum of points obtained from integrated models

NSL risk score (NE + SaO_2_ + LDH)		NL risk score (NE + LDH)	
Point of total	Estimate of risk of hospital mortality (%)	Point of total	Estimate of risk of hospital mortality (%)
0	0.18	0	0.22
1	0.25	1	0.33
2	0.33	2	0.49
3	0.45	3	0.74
4	0.61	4	1.09
5	0.82	5	1.62
6	1.10	6	2.40
7	1.48	7	3.54
8	1.99	8	5.20
9	2.67	9	7.56
10	3.57	10	10.87
11	4.76	11	15.39
12	6.32	12	21.35
13	8.34	13	28.82
14	10.94	14	37.66
15	14.22	15	47.40
16	18.29	16	57.35
17	23.20	17	66.73
18	28.97	18	74.95
19	35.50	19	81.70
20	42.63	20	86.94
21	50.07	21	90.85
22	57.52	22	93.68
23	64.63	23	95.67
24	71.16	24	97.06
25	76.91	25	98.01
26	81.80		
27	85.85		
28	89.12		
29	91.71		
30	93.72		
31	95.27		
32	96.45		
33	97.35		
34	98.02		

## Discussion

The NSL score and NL score described in this study are easy to understand and use. These two risk scores make it easy for clinicians to predict the risk of death in severe patients based on empirical data from patients and avoid the influence of personal bias in the course of evaluation. In some regions where medical resources are scarce, the NL score enables medical staffs to predict the risk of death of severe patients with only NE and LDH at the time of admission, which will greatly improve the efficiency of medical resource allocation and patient care. The NSL score and NL score were developed in a dataset of Chinese patients and validated in another dataset of Iranian patients. There were several differences in the clinical characteristics of the severe patients in the training and test datasets, but this suggests our risk scoring system is robust, as it provides similar predictability across these different patient populations.

Lymphopenia, neutrophilia, LDH, D-dimer and CRP may be related to the progression of COVID-19 disease according to previous studies [3–5, 7, 8]. Among these factors, elevated D-dimer and lymphopenia have been reported to be associated with death [3, 4, 7]. An SaO_2_ rate below 93% (normal range is 95% to 100%) has long been considered a sign of underlying hypoxia and impending organ failure [13, 14]. For COVID-19, SaO_2_ is also a good indicator for the disease progression [15], which is also confirmed by our models. A previous study found that higher sequential organ failure assessment (SOFA) score, older age, and D-dimer greater than 1 μg/mL at admission were associated with increased risk of death, which could help medical staffs assess the prognosis of patients [3]. In addition, Ji et al. established a risk score (CALL) based on patients’ age, lymphocyte count, serum LDH levels and comorbidities at admission, which could help medical staffs to identify patients with a high risk of disease progression [5]. Outside of the CALL risk score to predict risk of disease progression, clinicians lack a relevant scoring system to quantitatively predict the risk of death in severe patients. This may lead to an underestimation of the risk of death in some severe patients, resulting in delays in treatment and unnecessary mortality.

We utilized the feature selection method of machine learning and also considered the needs of clinicians to create our predictive models with the available data. We established two risk scores (NSL score and NL score) based on NE, SaO_2_ with and without LDH concentration at admission. An NSL score ≤ 11 is associated with a risk of death of less than 5%, whereas NSL score > 15 and particularly > 20 indicated an increased risk of death in patients; requiring urgent symptomatic treatment and careful surveillance for these patients. In particular, the cut-off point of 20 in NSL score offered 71% sensitivity and 94% specificity for death risk prediction in training datasets and 92% sensitivity and 82% specificity in the test dataset. For some regions without appropriate access to tests for SaO_2_ concentrations in patients, the NL score can also be used to predict the risk of death with high risk prediction accuracy. NL score ≤ 8 is associated with a risk of death of less than 5%, whereas NL score > 9 and NL score > 14 indicated the risk of death exceeding 10% and 40%, respectively.

Our study has a few limitations. First, the sample size is relatively small, especially the test dataset from Iran. Second, due to the limitations of data, we could not analyze the effects of different medical interventions on prognosis. Finally, the predictive capacity of the NSL and NL risk scores for the risk of death in patients with COVID-19 may be affected by the concentration of LDH and the proportion of patients with higher concentrations. In our study, the analyzer machines and methods used to determine serum LDH concentrations are different in China and Iran, and the normal range of LDH concentrations is slightly different. In China, LABOSPECT 008 α Hitachi Automatic Analyzer (Hitachi High-Technologies Corporation, Japan) was applied to detect serum LDH concentrations (normal range < 245 U/L), while in Iran, LDH Cytotoxicity Detection Kit (Roche, Germany) was used (normal range < 480 U/L). The serum LDH ranged from 121 to 1673 U/L in the Chinese cohort of patients with COVID-19 and the serum LDH ranged from 189 to 1642 U/L in the Iranian cohort. Although the concentration range of LDH was roughly the same in both cohorts, the proportion of patients with the concentration of LDH above 721 U/L in Iranian cohort was higher than that in Chinese cohort (37.2% vs. 9.4%), which may explain why the NSL and NL risk scores have higher specificity for predicting risk of death when using higher cut-off values (NSL > 20 and NL > 15), but significantly lower specificity when selecting lower cut-off values (NSL > 15 and NL > 12) in the Iranian cohort. In addition, we evaluated the predictive capacity of LDH, NE, and SaO_2_ for risk of death in the Iranian cohort. Obviously, the predictive capacity of LDH for the risk of death in the Iranian cohort was lower than that in the Chinese cohort (0.764 vs. 0.880), which was not found in predictive capacity of NE and SaO_2_. The predictive capacity of the NSL and NL risk scores for the risk of death in patients with COVID-19 may be affected by the concentration of LDH and the proportion of patients with higher concentrations. Therefore, clinicians should be cautious in using the NSL and NL risk scores, and large cohorts are still needed to test the predictive ability of these two risk models for mortality risk of patients with COVID-19.

## Conclusions

In conclusion, the NSL score and NL score, which are based only on two or three parameters of routine blood and biochemical tests at hospital admission, are straightforward objective approaches to predict the risk of death in severe COVID-19 patients, representing simple, reliable and widely applicable scores for predicting the mortality risk in severe COVID-19 patients.

## Supplementary Information

**Additional file** **1. Fig. S1**: Changes of baseline laboratory tests and the ability of integrated models to predict hospital mortality in severe patients. **Table S1**: Variables in risk models associated with hospital mortality in the training dataset of 96 severe patients with COVID-19. **Table S2**: Accuracy for prediction of hospital mortality of severe patients in the train and test datasets.

## Data Availability

Additional data are available from the first author and corresponding author upon reasonable request.

## Abbreviations

COVID-19: Coronavirus disease 2019
SARS-CoV-2: Severe acute respiratory syndrome coronavirus-2
NSL model: Neutrophil percentage, lactate dehydrogenase and oxygen saturation model
NL model: Neutrophil percentage and lactate dehydrogenase model
AUC: Area under the curve
SaO_2_: Oxygen saturation
ARDS: Acute respiratory distress syndrome
ALT: Alanine aminotransferase
AST: Aspartate aminotransferase
LDH: Lactate dehydrogenase
CRP: C-reactive protein
WBC: White blood cells
NE: Neutrophil percentage
LY: Lymphocyte percentage
HGB: Hemoglobin
HCT: Hematocrit
PLT: Platelets
Tbil: Total bilirubin
Dbil: Direct bilirubin
ALB: Albumin
APTT: Activated partial thromboplastin time
PT: Prothrombin time
BUN: Blood urea nitrogen
Cr: Serum creatinine
CCr: Creatinine clearance
CKMB: Creatine kinase isoenzymes
HDL: High density lipoprotein
LDL: Low density lipoprotein
TC: Total cholesterol
TG: Triglyceride
ApoA: Apolipoprotein A
ApoB: Apolipoprotein B
K: Serum potassium
Na: Serum sodium
SE: Standard error
ROC: Receiver operating characteristic
