# Supplementary material for: Scores based on neutrophil percentage and lactate dehydrogenase with or without oxygen saturation predict hospital mortality risk in severe COVID-19 patients
Source: Virol J. 2021 Mar 31;18:67. doi: 10.1186/s12985-021-01538-8 (PMC8011050; doi:10.1186/s12985-021-01538-8)
Supplement: Supplementary file 1 — Additional file 1. Fig. S1: Changes of baseline laboratory tests and the ability of integrated models to predict hospital mortality in severe patients. Table S1: Variables in risk models associated with hospital mortality in the training dataset of 96 severe patients with COVID-19. Table S2: Accuracy for prediction of hospital mortality of severe patients in the train and test datasets. [file 12985_2021_1538_MOESM1_ESM.doc]

**Supplementary Figure 1. Changes of baseline laboratory tests and the ability of integrated models to predict hospital mortality in severe patients**





Abbreviation: LDH, lactate dehydrogenase; NE, neutrophil percentage; SaO_2_, oxygen saturation; LY, lymphocyte percentage; NLR, neutrophils/lymphocytes ratio; CKMB, creatine kinase myocardial bound; CRP, C-reactive protein.

**Supplementary Table 1. Variables in risk models associated with hospital mortality in the training dataset of 96 severe patients with COVID-19.**

| Variables | Univariable analysis | | | Multivariable analysis | | | |
| --- | --- | --- | --- | --- | --- | --- | --- |
|  | OR | (95% CI) | P value | Bi | OR | (95% CI) | P value |
| **NSL risk score** |  |  |  |  |  |  |  |
| NE | 1.228 | 1.124-1.342 | <0.001 | 0.127 | 1.136 | 1.027-1.255 | 0.013 |
| SaO_2_ | 0.769 | 0.692-0.854 | <0.001 | -0.175 | 0.839 | 0.740-0.952 | 0.006 |
| LDH | 1.008 | 1.004-1.011 | <0.001 | 0.003 | 1.003 | 1.001-1.006 | 0.038 |
| **NL risk score** |  |  |  |  |  |  |  |
| NE | 1.228 | 1.124-1.342 | <0.001 | 0.158 | 1.171 | 1.065-1.288 | 0.001 |
| LDH | 1.008 | 1.004-1.011 | <0.001 | 0.004 | 1.004 | 1.001-1.008 | 0.017 |

Abbreviation: LDH, lactate dehydrogenase; NE, neutrophil percentage; SaO2, oxygen saturation; OR, odds ratio; CI, confidence interval; Bi, the regression coefficient.

**Supplementary Table 2. Accuracy for prediction of hospital mortality of severe patients in the train and test datasets**

| **Risk score** | **Training data**  **(N = 96)** | **Test data**  **(N = 43)** |
| --- | --- | --- |
| **NSL risk score** |  |  |
| AUC | 0.928(0.879-0.976) | 0.901(0.747-1.000) |
| **NSL risk score >15** |  |  |
| Sensitivity | 94 | 92 |
| Specificity | 82 | 39 |
| **NSL risk score >20** |  |  |
| Sensitivity | 71 | 92 |
| Specificity | 94 | 82 |
| **NL risk score** |  |  |
| AUC | 0.895(0.834-0.956) | 0.857(0.713-1.000) |
| **NL risk score >12** |  |  |
| Sensitivity | 94 | 92 |
| Specificity | 75 | 54 |
| **NL risk score >15** |  |  |
| Sensitivity | 65 | 92 |
| Specificity | 91 | 75 |

Abbreviation: AUC, area under the curve.
